# Identifying missed clinical opportunities for the earlier diagnosis of HIV in Australia, a retrospective cohort data linkage study

**DOI:** 10.1371/journal.pone.0208323

**Published:** 2018-12-06

**Authors:** Kylie-Ann Mallitt, David P. Wilson, James Jansson, Ann McDonald, Handan Wand, Jeffrey J. Post

**Affiliations:** 1 The Kirby Institute, University of New South Wales, Sydney, NSW, Australia; 2 Infectious Diseases, Prince of Wales Hospital, Sydney, NSW, Australia; 3 Prince of Wales Clinical School, University of New South Wales, Sydney, NSW, Australia; Katholieke Universiteit Leuven Rega Institute for Medical Research, BELGIUM

## Abstract

**Background:**

Treatment as prevention approaches for HIV require optimal HIV testing strategies to reduce undiagnosed HIV infections. In most settings, HIV testing strategies still result in unacceptably high rates of missed and late diagnoses. This study aimed to identify clinical opportunities for targeted HIV testing in persons at risk to facilitate earlier HIV diagnosis in New South Wales, Australia; and to assess the duration between the diagnosis of specific conditions and HIV diagnosis.

**Methods:**

The Australian National HIV registry was linked to cancer diagnoses, notifiable condition diagnoses, emergency department presentations and hospital admissions for all HIV diagnoses between 1993 and 2012 in NSW. Date of HIV acquisition was estimated from back-projection models and people with a likely duration from infection to diagnosis of less than 180 days were excluded. Risk factors associated with clinical opportunities for the earlier diagnosis of HIV were identified.

**Results:**

Sexually transmitted infection diagnoses (particularly gonorrhoea and syphilis) and some hospital admissions (mental health and drug-related diagnoses, and non-infective digestive disorder diagnoses) were prominent among people estimated to be living with undiagnosed HIV. The length of time between a clinical opportunity for the earlier HIV diagnosis and actual HIV diagnosis was 13.3 months for notifiable conditions, and 15.2 months for hospital admissions. People with lower CD4+ cell count at diagnosis, and older people were significantly less likely to have a missed opportunity for earlier HIV diagnosis.

**Conclusions:**

Additional targeted clinical HIV testing strategies are warranted for people with gonorrhoea and syphilis; and hospital presentations or admissions for mental health, drug-related and gastrointestinal diagnoses.

## Introduction

Antiretroviral therapy (ART) reduces the onward transmission of HIV, and global HIV prevention efforts are consequently focussed around treatment [1]. Modelling studies demonstrate that treatment as prevention approaches are optimised when a large proportion of people living with HIV (PLHIV) are diagnosed and on treatment [2]. In Australia, it is estimated that 11% of HIV infections were undiagnosed in 2016 [3]. Effective treatment is also delayed by the “late” diagnosis of HIV, which is associated with increased short-term mortality [4,5]. In Australia, 33% of people diagnosed with HIV in 2016 were diagnosed with a CD4 T-cell count below 350 cells/uL [6]. A key goal of UNAIDS is the 90-90-90 target, which aims to globally achieve 90% known serostatus status among PLHIV, 90% of diagnosed PLHIV receiving sustained ART, and 90% of people receiving ART to have viral suppression by 2020 [7,8].

To achieve this goal, there is a need to optimise HIV testing strategies to increase the number of PLHIV who are diagnosed and linked to treatment [2]. The main testing strategies utilised in developed countries involve targeted screening of at risk individuals. The British and Australian National HIV Testing Policies both indicate HIV testing based largely on indicator conditions and epidemiological risk-factors, such as sexual behaviour [9,10]. However, previous research has demonstrated that this approach is failing to detect many HIV infections [11–14]. Clinicians may fail to identify high-risk individuals based on their epidemiological characteristics, or patients may fail to disclose risk factors [15]. In addition, people from marginalised populations may not attend services where targeted screening typically takes place (such as sexual health or genitourinary medicine clinics) [16]. In Australia, HIV testing can be accessed through general practitioners, sexual health clinics, and hospitals. Rapid testing can also be accessed in clinical settings and through some community organisations. HIV rapid tests are not currently approved by the Therapeutic Goods Association for home use. In the clinical setting, HIV testing is not undertaken in all cases of viral hepatitis and late HIV diagnosis has been documented [17]. People without disclosed or unrecognised HIV risk presenting with primary HIV infection may be misdiagnosed with other viral infections [18].

An alternative testing strategy is universal HIV screening, which has been investigated widely in the United States and trialled in several other countries [19]. Recommendations have been made for overall HIV screening of the general population, as well as routine screening in hospital emergency departments [20]. These have been successful in some local situations, but unsuccessful on a large scale [21]. This is largely due to the burden of universal testing on the health care system [22].

The clinical indicators for HIV testing in adults includes a range of infections, cancers and other clinical conditions, some of which are associated with hospital encounters [10]. More highly focussed clinical, rather than epidemiological, risk assessment may be an approach to increase the opportunity for targeted HIV testing in persons at risk. Clinical encounters that temporally occur between the estimated date of HIV acquisition and the date of HIV diagnosis (i.e. undiagnosed HIV), provide a window of opportunity for the earlier diagnosis of HIV. The aims of the study were to: a) identify opportunities for health care contact where an HIV test may have led to an earlier HIV diagnosis; b) assess the duration between the diagnosis of specific conditions and HIV diagnosis; and c) identify access points for HIV testing to enable earlier HIV diagnosis in NSW.

## Materials and methods

We conducted a retrospective cohort study of people diagnosed with HIV in NSW, Australia, from 1993–2012. We collected data on cancer diagnoses (1994–2009), notifiable condition diagnoses (1993–2012), hospital admissions (2000–2012), and emergency department presentations (2005–2012), that occurred between the estimated time of HIV acquisition and HIV diagnosis. These databases were chosen to capture presentations with HIV indicator conditions [9,10], and to identify common non-indicator health system presentations or diagnoses that are not currently recognised as co-occurring with HIV. Notifiable conditions are a group of over 60 serious conditions, mostly communicable diseases, that diagnosing clinicians must report to the state health authority by law [6]. Data were made available through the linkage of routinely collected administrative datasets, which was conducted by The Centre for Health Record Linkage (CHeReL). Linked records were obtained from the following databases: the National HIV Registry; the NSW Admitted Patient Data Collection (APDC); the NSW Emergency Department Data Collection (EDDC); the NSW Central Cancer Registry; and the NSW Notifiable Conditions Information Management System (NCIMS). Data linkage was conducted using a 2-letter first name code, a 2-letter surname code, gender and date of birth. Diagnoses were analysed by International Classification of Disease (ICD) version 10 codes and presented by code or sub-chapter. The APDC was coded by professional medical coders, while the EDDC was coded by clinicians. Due to the time frame required for approval, record linkage and access to linked administrative data in Australia, the dataset used in this study (end 2012) was the most recent data available during the timeframe of this study.

Mathematical back-projection models were used to infer the date of HIV acquisition from the CD4 T cell count at diagnosis, and the date of diagnosis [23]. In brief, this method used data on the distribution of CD4 counts in a healthy population, and the rate of CD4 decline due to untreated HIV infection. The CD4 decline rate was used to back-project a likely distribution of time of infection for the CD4 count of each individual around the time of diagnosis. This gave a probability distribution of the year of infection for each individual. The median of these dates was used as the estimated date of acquisition, and was modified to take into account the date of known previous negative HIV tests as described by Jansson et al [22].

The inclusion criteria for the study were adults (18 years and older) diagnosed with HIV in NSW between 1993 and 2012. Study exclusion criteria were previous overseas diagnosis of HIV infection; clinical evidence of HIV infection in the admitted patient data collection; and evidence of HIV acquisition within 180 days of HIV diagnosis. Data from 319 people in the National HIV Registry were matched to the cancer, notifiable condition, hospital admission or emergency department records of 682 people by CHeReL. Where one individual in the National HIV Registry was matched to the health records of two people by CHeReL, we used a random number generator to randomly exclude one of those records.

Cancer diagnoses, notifiable condition diagnoses, hospital admissions and emergency presentations that were linked to the HIV database (referred to subsequently as linked health care events), were tabulated for the most frequent linked health care event diagnoses. For hospital admissions, the number of hospital admissions with a diagnosis that met the Australian clinical indicators for HIV testing criteria was determined [10].

The rate of linked health care events in the population of people with undiagnosed HIV was compared to the rate of the same events in the general NSW population using indirectly standardised incidence ratios (and 95% confidence intervals). Background rates of defined health care events were obtained from publicly-available published sources where suitable data were available [24–27]. Based on the linkage results, measures of efficiency associated with the introduction of routine testing with each access point were calculated. The ‘number needed to test (NNT)’ was reported, which is the number of people diagnosed with a linked health care event in the general NSW population that would need to be tested for HIV to achieve one additional HIV diagnosis (assuming 100% sensitivity of the HIV test). Demographic factors associated with the time to linked health care events were assessed by multivariate Cox proportional hazards regression analysis. For each linked health care database, patients were followed from the beginning of data records (1994, 1993, 2000, and 2005 for cancer, notifiable conditions, hospital admissions emergency department presentations, respectively). Follow-up ceased at the end of 2009 for cancer diagnoses, and 2012 for the analyses of the other linked databases. The analysis was adjusted for CD4 count at diagnosis, age, sex, country of birth, HIV mode of transmission, metropolitan/regional residence, and socio-economic status of residential location (measured by SEIFA -Socioeconomic Index for Areas).

The number of linked health care events were summarised by Local Health District (LHD) of diagnosis or presentation. LHDs are health-services administrative boundaries that divide NSW into 15 geographical regions. The rate of missed HIV diagnoses at the time of linked health care events were calculated as the number of linked health care events during the time period covered by each linked database divided by either the number of PLHIV, or the number of people in the general population, during the same time period. These were calculated as the rate per 1,000 person-years for PLHIV, and per 100,000 person-years for the general population. These rates were mapped by LHD. The number of person-years for people in the general population of NSW residing in each LHD was obtained from Health Statistics NSW [28] and the Australian Bureau of Statistics [24]. The number of PLHIV in each LHD was estimated using mathematical modelling in a previous study [29]. Briefly, an agent-based simulation model was linked to the Australian National Registry of HIV diagnoses and, in combination with data on internal migration patterns from the Australian Bureau of Statistics and Australian population mortality rates, was used to estimate the size of the population of PLHIV in Australia by LHD. All statistical analyses were conducted in SAS v9.3, and all spatial analyses were conducted in ArcGIS v10. Ethical approval for the study was obtained from the New South Wales Population and Health Services Research Ethics Committee (Reference: HREC/13/CIPHS/59). The data were fully anonymised prior to access by the authors of this study, and thus written informed consent was not sought from patients.

## Results

In NSW, 8572 people were diagnosed with HIV infection between 1993 and 2012, of whom 6430 were eligible for inclusion in this study. The median time from estimated HIV acquisition to HIV diagnosis was 3.3 years. Overall, 42.4% of people had a missing CD4 cell count at HIV diagnosis; however, this comprised mostly people diagnosed with HIV infection prior to 2008. There were 27.7% and 29.9% of people diagnosed with HIV infection with a CD4 cell count less than 200 cells per uL (very late diagnosis), or less than 350 cells per uL (late diagnosis), respectively.

Twenty-eight percent of people were estimated to have acquired their HIV infection between 1982 and 1992 (Table 1). Of the 6430 people in the study, 57 (0.9%) had 58 cancer diagnoses, 379 (5.9%) had 440 notifiable condition diagnoses, 639 (9.9%) had 1415 hospital admissions, and 653 (10.2%) had 1991 emergency department presentations between the estimated time of HIV infection and HIV diagnosis (Table 2). HIV diagnoses tended to occur very rapidly following a cancer diagnosis (0.7 months); while the duration between each linked health-care event and HIV diagnosis was much longer for notifiable conditions, hospital admissions and emergency department presentations (ranging from 13.3 to 16.6 months). Compared to the general population of NSW, people living with undiagnosed HIV infection were 3.6 times more likely to be diagnosed with any cancer (95% CI 2.7–4.5), and 1.6 times more likely to have a hospital admission (95% CI 1.5–1.6).

**Table 1:** Number of NSW HIV diagnoses by year interval of diagnosis and estimated date of HIV acquisition (1993–2012).

	Year of HIV Diagnosis			
	1993–2004	2005–2008	2009–2012	Total
**Total**	4378	1130	922	6430
**Estimated Year of HIV Acquisition**				
1982–1992	1812	0	0	1812
1993–2004	2566	749	175	3490
2005–2008	-	381	401	782
2009–2012	-	-	346	346

**Table 2:** Number of health care events linked to NSW HIV diagnoses for cancer diagnoses, notifiable conditions, hospital admissions and emergency department presentations (1993–2012).

	Cancer Diagnoses^1^ (n)	Notifiable Condition Diagnoses^2^ (n)	Hospital Admissions^3^ (n)	Emergency Department Presentations^4^ (n)
Number of people with an event	57	379	639	653
Number of total events	58	440	1415	1991
Number of events per person 1	56	328	360	287
2–3	1	51	185	204
4–9	-	-	87	135
10+	-	-	7	27
Time from Event to HIV Diagnosis months(Median (IQR))	0.7 (9.0)	13.3 (24.4)	15.2 (26.9)	16.6 (33.1)
Standardised Incidence Ratio^5^ (95% CI)	3.6 (2.7–4.5)	-	1.6 (1.5–1.6)	-
Number Needed to Test^6^	9,017	-	22,540	7921

^1^Cancer diagnosis data was available from 1994–2009.

^2^Notifiable condition diagnosis data was available from 1993–2012.

^3^Hospital admission data was available from 2000–2012.

^4^Emergency department presentation data was available from 2005–2012.

^5^Standardised incidence ratio relative to the general population of NSW.

^6^Number of people with a linked health care event needed to be tested for HIV to achieve one additional HIV diagnosis (assuming 100% sensitivity of HIV test).

The most common cancer diagnosed was Kaposi sarcoma (n = 22; 37.9%) (Table 3). Almost half of the cancers were diagnosed in the period 1994–1997 (n = 27; 46.6%). The most common notifiable condition diagnosed was gonorrhoea (n = 159; 36.1%) (Table 3). All common notifiable conditions were blood-borne and/or sexually transmitted infections. The period in which the most notifiable conditions were diagnosed was 2001–2004 (n = 138; 31.4%). From 2009 to 2012, the rate of gonorrhoea diagnosis was 18.0 times higher among people with undiagnosed HIV than in the general population of NSW (95% CI 8.2–27.8). The median time from gonorrhoea diagnosis to HIV diagnosis was 14.4 months. One hundred and seventy-eight people diagnosed with gonorrhoea needed to be tested for HIV to diagnose one additional case of HIV infection. The median time from syphilis diagnosis to HIV diagnosis was 11.7 months. Four hundred and forty-four people diagnosed with syphilis needed to be tested for HIV to diagnose one additional case of HIV infection.

**Table 3:** Number of health care events in NSW linked to people with subsequent HIV diagnosis by year interval of linked health care event diagnosis and type of a) cancer diagnosis (1994–2009); b) notifiable condition diagnosis (1993–2012); c) hospital admission diagnosis (2000–2012); and d) emergency department presentation diagnosis (2005–2012).

Diagnosis	Total
**All Cancers** (ICD10 code)	58
Kaposi sarcoma (C46)	22
Non-Hodgkin's lymphoma (C82)	12
Malignant melanoma of skin (C43)	6
Malignancy of rectum, anus (C19-C21)	3
Other*	15
**All Notifiable Conditions**	440
Gonorrhoea	159
Hepatitis C	72
Chlamydia^	66
Hepatitis B	31
Syphilis	25
Hepatitis A	23
Other	64
*Any STI*^*#*^	*250*
*Hepatitis B or C*	*103*
**All Hospital Admissions** (ICD10 code subchapter)	1415
Mental/behavioural disorders due to psychoactive substance (F10-F19)	82
Other diseases of intestines (K55-K64)	80
*Irritable bowel syndrome/functional intestinal disorders (K58-K59)*	*11*
*Fissure*, *fistula*, *abscess or other disorder of anus or rectum (K60-K64)*	*49*
Other dorsopathies (M50-M54)	71
Diseases of oesophagus, stomach and duodenum (K20-K31)	41
Influenza and pneumonia (J09-J18)	39
Infections of the skin and subcutaneous tissue (L00-L08)	36
Symptoms/signs involving the digestive system and abdomen (R10-R19)	36
Persons encountering health services for specific procedures (Z40-Z54)	34
Poisoning by drugs, medicaments and biological substances (T36-T50)	33
Symptoms/signs involving the circulatory/ respiratory systems (R00-R09)	32
General symptoms and signs (R50-R69)	32
Schizophrenia, schizotypal and delusional disorders (F20-F29)	30
Non-infective enteritis and colitis (K50-K52)	29
Other	836
**All Emergency Department Presentations**	1991
Refusal of treatment	51
Other physical trauma or injury	40
Pneumonia	22
Mental health or drug issue	18
Other respiratory infections	16
Abdominal pain	12
Gastroenteritis and colitis	10
Genital/anal trauma or injury	10
Chest pain	9
Other	1322
Missing	481

* Other cancers include bladder, larynx, testis, ill-defined & unspecified site, brain, central nervous system, cervix uteri, colon, kidney, lip, prostate, salivary glands

^ Chlamydia was a notifiable condition in New South Wales from 1998

^#^ Any STI includes chlamydia, gonorrhoea or syphilis

The most common principal diagnosis for hospital admission was ‘mental/behavioural disorders due to psychoactive substance’ (n = 82) (Table 3). Four main principal diagnosis themes were observed among people with undiagnosed HIV who were admitted to hospital 1) Mental health and drug use: ICD-10 codes F10-F19, F20-F29, T36-T50; 2) Digestive disorders: ICD-10 codes K55-K64, K20-K31; K50-K52; 3) Respiratory disorders: ICD-10 codes J09-J18; and 4) Symptomatic diagnoses (admissions where a specific diagnosis was not made): ICD-10 codes R00-R09, R10-R19, R50-R69. Of the 1415 hospital admissions, 103 (7.3%) had a diagnosis that matched current Australian clinical indicators for HIV testing. Among all hospital admissions, 6.1% (n = 87) were admitted to a psychiatric unit. The rate of admission did not change significantly over time (p = 0.173), however, was significantly more likely among those diagnosed with HIV with a missing CD4 cell count (p<0.0001). People diagnosed with mental/behavioural disorders due to psychoactive substance use were 5.3 times more likely to be admitted (95% CI 4.2–6.5). To diagnose one additional case of undiagnosed HIV infection 22,540 people admitted to hospital needed to be tested for HIV (Table 2). However, only 2,803 people diagnosed with mental/behavioural disorders due to psychoactive substance needed to be tested for HIV to diagnose one additional case of HIV infection.

The most common emergency department primary diagnoses among the population of undiagnosed PLHIV was ‘refusal of treatment’ (n = 51) (Table 3). This may be indicative of the ‘difficult-to-reach’ nature of the population, with a high degree of mental health and drug-use related hospital admissions. Mental health or drug issues, respiratory conditions (pneumonia), and non-infective digestive conditions were also among the most common emergency department diagnoses.

People with undiagnosed HIV infection with a missing or low CD4 count (<350 cells/uL) had a 29% and 34% reduced rate of linked notifiable condition diagnosis whilst undiagnosed with HIV than people with a CD4 count over 350 cells per uL (Table 4). Women had an 88% higher rate of notifiable condition diagnosis whilst undiagnosed with HIV than men. People with undiagnosed HIV infection with a low CD4 count (<350 cells/uL) had a 29% lower rate of hospital admissions whilst undiagnosed with HIV than people with a CD4 count over 350 cells per uL (Table 4). For every 10 years of age, people had a 25% lower rate of hospital admission whilst undiagnosed with HIV People with undiagnosed HIV infection with a missing CD4 count had a 3.74 times higher rate of emergency department presentation than people with a CD4 count over 350 cells per uL (Table 4). For every 10 years of age, people had a 17% higher rate of emergency department presentation whilst undiagnosed with HIV. Females had a 34% higher rate of emergency department presentation whilst undiagnosed with HIV than men.

**Table 4:** Demographic risk factors for the time to first notifiable condition, hospital admission or emergency department presentation among people with HIV diagnoses in NSW, 1993–2012, after the date of estimated HIV acquisition and before HIV diagnosis.

		Notifiable Conditions	Hospital Admissions	Emergency Presentations
Variable		Multivariate HR (95% CI)	Multivariate HR (95% CI)	Multivariate HR (95% CI)
CD4 Count at Diagnosis	350+	1	1	1
	0–349	0.66 (0.49–0.89)	0.71 (0.56–0.89)	1.00 (0.89–1.36)
	Missing	0.71 (0.53–0.95)	1.41 (1.07–1.85)	3.74 (2.88–4.85)
Sex	Male	1	-	1
	Female	1.88 (1.18–3.00)	-	0.66 (0.49–0.90)
Age at HIV (years)	Per 10 years	-	0.75 (0.68–0.81)	0.83 (0.76–0.91)
HIV Exposure	MSM	-	1	-
	Other	-	0.57 (0.46–0.70)	-
Country of Birth	Australia	1	1	-
	Other	1.48 (1.15–1.90)	1.44 (1.51–1.80)	-
RRMA*	Metropolitan	-	1	
	Regional	-	0.71 (0.51–0.99)	
SEIFA^^^ Score	Per 10 points	-	-	1.03 (1.01–1.04)

* RRMA–Rural, Remote, Metropolitan Area classification

^ SEIFA—Socio Economic Indexes for Areas

**Fig 1:**
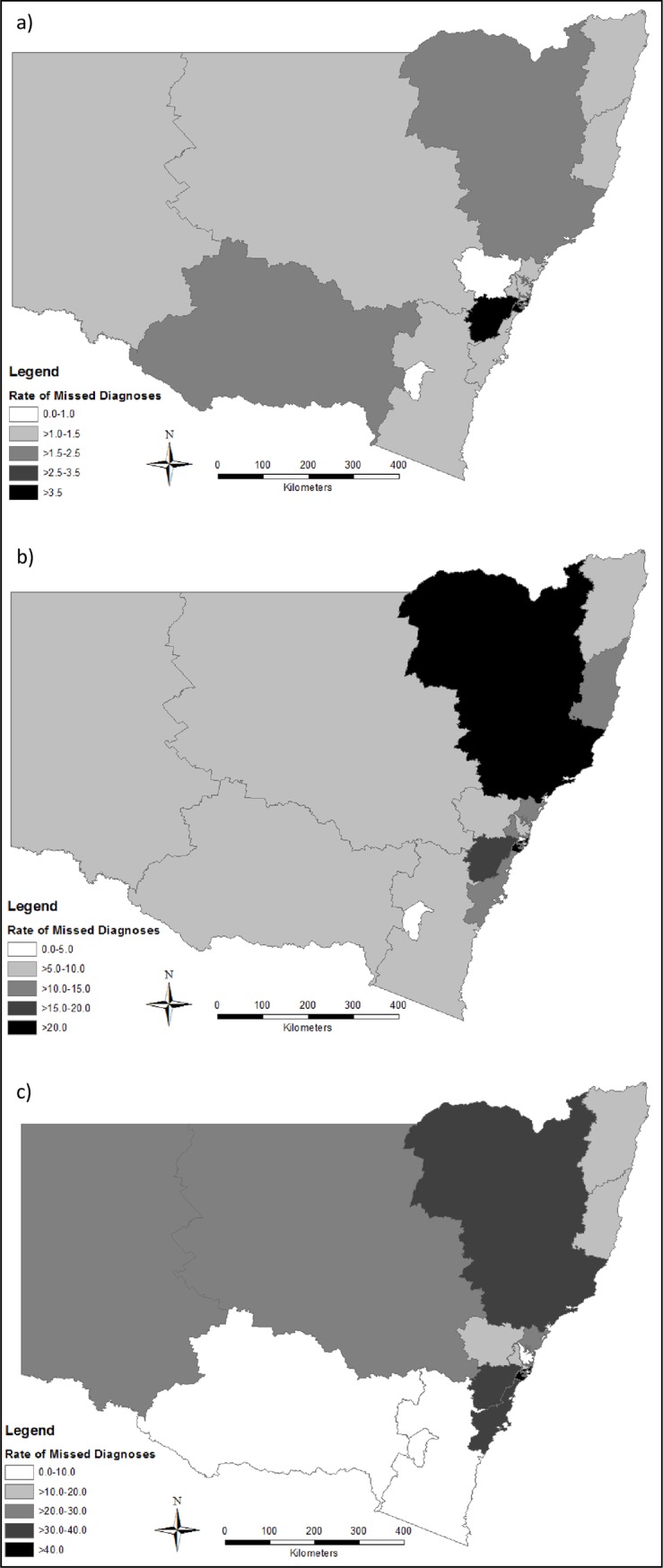
Rate of missed opportunities for a diagnosis of HIV due to a) notifiable condition diagnosis; b) hospital admission; c) emergency department presentation, per 1000 person-years for people living with HIV (1993–2012), by Local Health District of residence.

**Fig 2:**
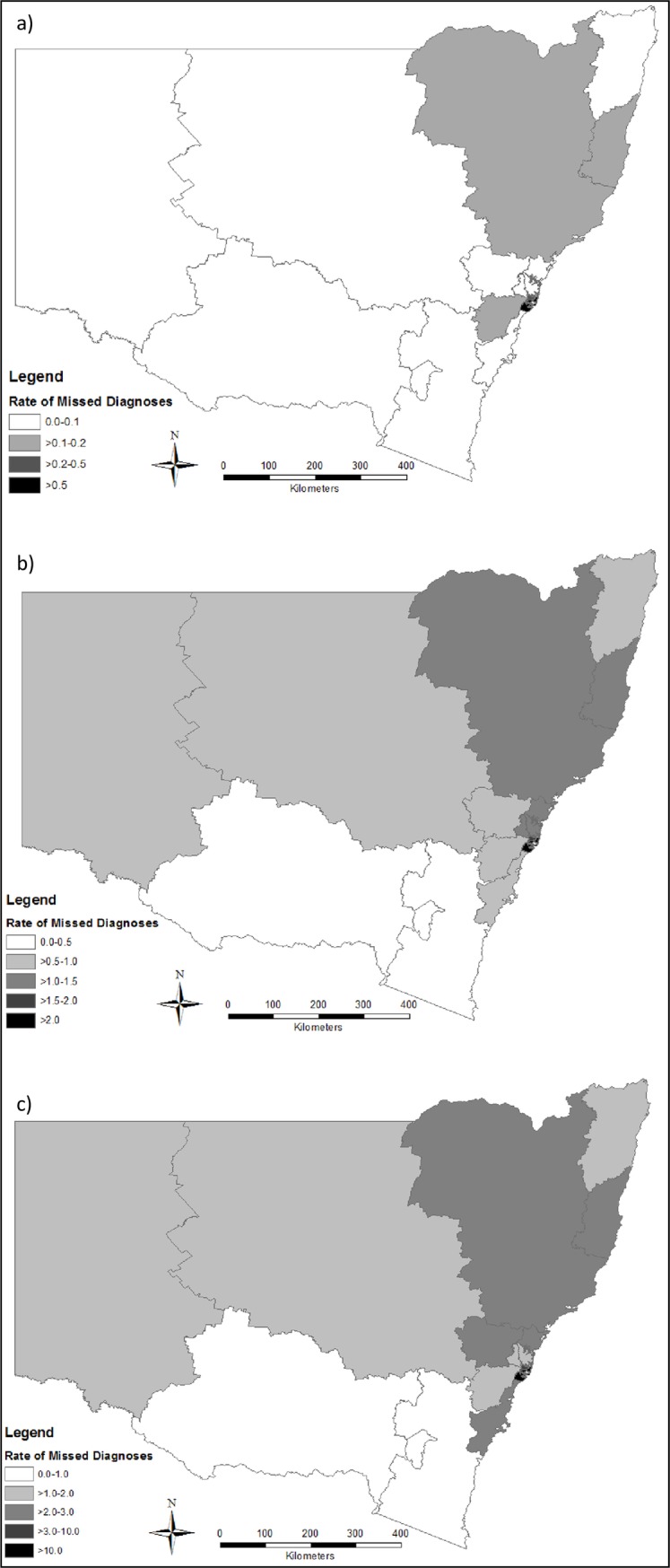
Rate of missed opportunities for a diagnosis of HIV due to a) notifiable condition diagnosis; b) hospital admission; c) emergency department presentation, per 100,000 person-years for the general population (1993–2012), by Local Health District of residence.

The rate of missed opportunities for HIV diagnosis at the time of a notifiable condition diagnosis (1993–2012) was 2.5 per 1000 person-years for people living with HIV in NSW; and 0.3 per 100,000 person-years for the general population. South Eastern Sydney LHD had highest rate of missed opportunities, at 7.6 per 1000 person-years for PLHIV, and 1.5 per 100,000 population (Figs 1A and 2A). The rate of missed opportunities for HIV diagnosis at the time of a hospital admission was 11.7 per 1000 person-years for people living with HIV in NSW; and 1.6 per 100,000 person-years for the general population (Figs 1B and 2B). The rate of missed opportunities for HIV diagnosis at the time of an emergency department presentation was 25.4 per 1000 person-years for people living with HIV in NSW; and 3.6 per 100,000 person-years for the general population (Figs 1C and 2C).

## Discussion

We conducted data linkage of routinely-collected administrative health datasets to identify clinical opportunities for increased HIV testing, to facilitate the earlier diagnosis and treatment of HIV. The date of HIV acquisition was estimated to identify occasions of contact with a clinical health service where a HIV infection diagnosis was potentially ‘missed’. We found that additional strategies to enhance testing are warranted for people with specific notifiable condition and hospital admission diagnoses.

Among people who are estimated to have acquired an HIV infection, there is a large window of opportunity for the earlier diagnosis of HIV at the time of a notifiable condition diagnosis. The median time from notifiable condition diagnosis to HIV diagnosis was 13.3 months, and 80% of these notifiable conditions were sexually transmissible infections or viral hepatitis. Particular opportunities were apparent for gonorrhoea and syphilis. There were very high numbers of gonorrhoea and syphilis diagnoses in the population of people with undiagnosed HIV relative to the general population of NSW (56 times and 24 times higher, respectively). Targeted testing for HIV among people diagnosed with gonorrhoea will offer the largest return on investment, with 178 people needed to be tested to achieve one additional HIV diagnosis. Gonorrhoea diagnoses are increasing in Australia [6], particularly among MSM. However, rates of gonorrhoea are relatively stable in the US [30]. Gonorrhoea is becoming the focus of international attention due to the rise in drug-resistant strains [31]. A study conducted in Florida found that women with gonorrhoea and syphilis were at high risk of HIV [32]. It is well established that primary care physicians do not consistently test for HIV among people with indicator conditions [4,33,34]. In an Australian study, 42.6% of patients with a late diagnosis of HIV had previous indicator conditions [34]. In the UK, less than half of people diagnosed with Hepatitis B and C were tested for HIV within six months of diagnosis [4]. In a Dutch study, the number of visits to primary care was frequent in the years before HIV diagnosis [35]. Diagnosis with an STI is evidence of sexual risk taking, with an increased likelihood of co-infection. Concurrent testing for HIV is a cost-effective approach. Our results indicate that there would be benefit from increased targeted clinical HIV testing for people diagnosed with STIs and viral hepatitis in the primary care setting.

We also identified clinical opportunities for the earlier diagnosis of HIV during hospital presentations and admissions. There was a median of 15.2 months between hospital admissions and HIV diagnosis, providing significant opportunity for the earlier detection of HIV. Hospital presentations and admissions among people with undiagnosed HIV are associated with diagnoses for drug use related mental health conditions, non-specific intestinal diagnoses, some respiratory disorders, and symptomatic diagnoses. Across all admissions, 7.3% had a diagnosis that matched current Australian clinical indicators for HIV testing, particularly fever of unknown origin and ill-defined diarrhoea. Also, 100 admissions (7.1%) had symptomatic diagnoses rather than definitive diagnoses. This may in-part reflect a lack of awareness among hospital clinicians around clinical indicators for HIV testing [36,37]. We also identified 150 patients with gastrointestinal-related diagnoses during admission. This is consistent with undiagnosed HIV infection. Almost half of HIV-infected patients present with frequent, and often nonspecific, gastrointestinal symptoms such as anorexia, weight loss, dysphagia, odynophagia, abdominal pain, and diarrhoea [38].

In our study, over 10% of admissions were for mental health or drug-related diagnoses and 6.1% of admissions were to the psychiatric unit. HIV, mental illness and drug abuse are a recognised ‘syndemic’; or group of linked health problems that occur when two or more afflictions interact synergistically and contribute to the excess burden of disease [39]. In a study of acute-care psychiatric hospital inpatients in the USA, 7.8% of admissions were HIV-related; and nearly half of these were patients experiencing functional or psychological complications of HIV infection or risk [40]. In our study, the highest burden of mental health admissions was due to schizophrenia (n = 22). Of drug-related behavioural admissions, most were also related to alcohol use. Gay men in Australia have been shown to have moderately-high to high levels of alcohol misuse [41]. We found that most non-alcohol drug-related admissions in our study were due to non-opioid stimulant use. Use of illicit stimulants, primarily cocaine and methamphetamine, has been associated with substantial amounts of HIV-related sexual risk behaviours when under the influence [42]. Relative to the general population, Australian MSM living with HIV are 2.5 times, and 3.5 times, more likely to be hospitalised for anxiety/mood disorders [43], and for drug related presentations [44], respectively. A recent study identified that blood borne virus testing for people presenting to two hospitals in south eastern Sydney for a drug related presentation was only undertaken in 12.9% of cases [45]. A routine offer of HIV testing should be undertaken for people who present to hospital or are admitted for a) drug use related mental health conditions and other drug use related diagnoses; b) undiagnosed intestinal symptoms or non-specific intestinal diagnoses; c) anal pathology (e.g. fissure, fistula, or abscess) or who are undergoing anal surgery.

In this study we have identified clinical opportunities to increase HIV testing among populations of people who are ‘difficult to reach’ in traditional testing settings. We found that among people with undiagnosed HIV, people born overseas and those with a missing CD4 T cell count at diagnosis are more likely to be admitted to hospital prior to their HIV diagnosis In addition, there were a number of admissions among people with mental health and drug abuse-related diagnoses. Individuals in these risk categories are less likely to seek out routine HIV testing [16,46,47]. There were a high proportion of people in our sample who left the emergency department prior to treatment. Previous research has demonstrated that individuals who do not consent to HIV tests in the emergency department are 2.7 times more likely to be HIV positive than those who are tested [48]. The Australian National HIV Testing Policy is highly focussed on ensuring voluntary testing with informed consent [10]. The Australian National HIV Strategy [49], NSW HIV Strategy [50], and UNAIDS goals [7] all strongly emphasise increased HIV testing. Future research is required around interventions to encourage the uptake of HIV tests among the at-risk target populations identified in this study.

A major strength of this study is the use of data linkage to provide a comprehensive follow-up of all cancer diagnoses, notifiable condition diagnoses, hospital admissions and emergency department presentations among all people diagnosed with HIV in NSW over a period of 8 to 20 years. This study has several limitations. The date of acquisition of HIV is estimated from a mathematical model. However, as some of the matched datasets had data available only after the period of probable infection acquisition for some of the study subjects, it is possible that the number of episodes detected has been underestimated. The full name of PLHIV was not available for matching for reasons of confidentiality. Previous studies show that when record linkage is conducted using a 2-letter first name code and 2-letter surname code, the number of records linked is an underestimate of the true number [51]. However, the random exclusion of 50% of records from the National HIV Registry that were matched to multiple health records by CHeReL is a minor source of error in the data. The data on presentations to the emergency department was coded inconsistently between 2005 and 2012, and in a less robust manner than the admissions data. This led to some difficulties in the interpretation of emergency diagnoses over time. However, the results from the emergency department data are broadly consistent with the results from the admissions data. The study excluded people with a likely window between infection and diagnosis of less than 180 days (n = 1758) and therefore is likely to have underestimated missed primary HIV infection presentations and other presentations, so that the NNTs presented here may be lower. The median duration between time of likely HIV acquisition and HIV diagnosis would also be lower if subjects were included within the 180-day window utilised in this study.

### Conclusions

Additional strategies are warranted to enhance HIV testing for people with notifiable condition diagnoses (particularly gonorrhoea and syphilis), and hospital admissions for mental health, drug related and gastrointestinal diagnoses. Additional testing strategies for people diagnosed with cancer do not appear to be warranted. Increased education around indications for HIV testing is needed for clinicians working in the fields of drug and alcohol, psychiatry, emergency medicine, toxicology, gastroenterology, colorectal and general surgery. Other non-educational strategies to enhance testing for people with such presentations are needed and these may include audit, feedback, task substitution, policy change and other strategies [45,46].

## Supporting information

S1 Table Number of health care events in NSW linked to people with subsequent HIV diagnosis by year interval of linked health care event diagnosis and type of a) cancer diagnosis (1994–2009); b) notifiable condition diagnosis (1993–2012); c) hospital admission diagnosis (2000–2012); and d) emergency department presentation diagnosis (2005–2012).
(DOCX)

S2 Table Number of NSW notifiable condition diagnoses linked to NSW HIV diagnoses, 1993–2012, and rate of missed opportunities per 1000 population of people living with HIV (PLHIV), by Local Health District of residence at diagnosis. (DOCX)

S3 Table Number of NSW hospital admissions linked to NSW HIV diagnoses, 2000–2012 and rate of missed opportunities for HIV diagnosis per 1000 person-years for population of people living with HIV (PLHIV), by Local Health District of admission. (DOCX)

S4 Table Number of NSW ED presentations linked to NSW HIV diagnoses, 2005–2012, and rate of missed opportunities per 1000 person-years for people living with HIV (PLHIV), by Local Health District of ED presentation. (DOCX)
