# Supplementary material for: Identifying missed clinical opportunities for the earlier diagnosis of HIV in Australia, a retrospective cohort data linkage study
Source: PLoS One. 2018 Dec 6;13(12):e0208323. doi: 10.1371/journal.pone.0208323 (PMC6283600; doi:10.1371/journal.pone.0208323)
Supplement: S1 Table — Number of health care events in NSW linked to people with subsequent HIV diagnosis by year interval of linked health care event diagnosis and type of a) cancer diagnosis (1994–2009); b) notifiable condition diagnosis (1993–2012); c) hospital admission diagnosis (2000–2012); and d) emergency department presentation diagnosis (2005–2012). (DOCX) [file pone.0208323.s001.docx]

**Supporting Information**

**S1 Table**

**Number of health care events in NSW linked to people with subsequent HIV diagnosis by year interval of linked health care event diagnosis and type of a) cancer diagnosis (1994-2009); b) notifiable condition diagnosis (1993-2012); c) hospital admission diagnosis (2000-2012); and d) emergency department presentation diagnosis (2005-2012).**

| **Diagnosis** | **1993-2004** | **2005-2008** | **2009-2012** | **Total** |
| --- | --- | --- | --- | --- |
| **All Cancers** (ICD10 code) | 44 | 11 | 3 | 58 |
| Kaposi sarcoma (C46) | 16 | 5 | 1 | 22 |
| Non-Hodgkin's lymphoma (C82) | 9 | 3 | 0 | 12 |
| Malignant melanoma of skin (C43) | 6 | 0 | 0 | 6 |
| Malignancy of rectum, anus (C19-C21) | 3 | 0 | 0 | 3 |
| Other* | 10 | 3 | 2 | 15 |
| **All Notifiable Conditions** | 321 | 83 | 36 | 440 |
| Gonorrhoea | 125 | 21 | 13 | 159 |
| Hepatitis C | 64 | 6 | 2 | 72 |
| Chlamydia^ | 39 | 19 | 8 | 66 |
| Hepatitis B | 25 | 5 | 1 | 31 |
| Syphilis | 7 | 15 | 3 | 25 |
| Hepatitis A | 23 | 0 | 0 | 23 |
| Other | 38 | 17 | 9 | 64 |
| *Any STI^#^* | 171 | 55 | 24 | *250* |
| *Hepatitis B or C* | 89 | 11 | 3 | *103* |
| **All Hospital Admissions** (ICD10 code subchapter) | 633 | 507 | 275 | 1415 |
| Mental/behavioural disorders due to psychoactive substance (F10-F19) | 41 | 23 | 18 | 82 |
| Other diseases of intestines (K55-K64) | 31 | 28 | 21 | 80 |
| *Irritable bowel syndrome/functional intestinal disorders (K58-K59)* | *4* | *7* | *0* | *11* |
| *Fissure, fistula, abscess or other disorder of anus or rectum (K60-K64)* | *19* | *12* | *18* | *49* |
| Other dorsopathies (M50-M54) | 15 | 46 | 10 | 71 |
| Diseases of oesophagus, stomach and duodenum (K20-K31) | 19 | 10 | 12 | 41 |
| Influenza and pneumonia (J09-J18) | 16 | 10 | 13 | 39 |
| Infections of the skin and subcutaneous tissue (L00-L08) | 17 | 14 | 5 | 36 |
| Symptoms/signs involving the digestive system and abdomen (R10-R19) | 13 | 16 | 7 | 36 |
| Persons encountering health services for specific procedures (Z40-Z54) | 18 | 12 | 4 | 34 |
| Poisoning by drugs, medicaments and biological substances (T36-T50) | 13 | 14 | 6 | 33 |
| Symptoms/signs involving the circulatory/ respiratory systems (R00-R09) | 16 | 13 | 3 | 32 |
| General symptoms and signs (R50-R69) | 14 | 7 | 11 | 32 |
| Schizophrenia, schizotypal and delusional disorders (F20-F29) | 15 | 5 | 10 | 30 |
| Non-infective enteritis and colitis (K50-K52) | 12 | 11 | 6 | 29 |
| Other | 393 | 298 | 149 | 836 |
| **All Emergency Department Presentations** | - | 1325 | 666 | 1991 |
| Refusal of treatment | - | 4 | 47 | 51 |
| Other physical trauma or injury | - | 3 | 37 | 40 |
| Pneumonia | - | 1 | 21 | 22 |
| Mental health or drug issue | - | 1 | 17 | 18 |
| Other respiratory infections | - | 1 | 15 | 16 |
| Abdominal pain | - | 0 | 12 | 12 |
| Gastroenteritis and colitis | - | 4 | 6 | 10 |
| Genital/anal trauma or injury | - | 1 | 9 | 10 |
| Chest pain | - | 2 | 7 | 9 |
| Other | - | 901 | 421 | 1322 |
| Missing | - | 407 | 74 | 481 |
