# Supplementary material for: Identifying missed clinical opportunities for the earlier diagnosis of HIV in Australia, a retrospective cohort data linkage study
Source: PLoS One. 2018 Dec 6;13(12):e0208323. doi: 10.1371/journal.pone.0208323 (PMC6283600; doi:10.1371/journal.pone.0208323)
Supplement: S2 Table — (DOCX) [file pone.0208323.s002.docx]

**S2 Table**

**Number of NSW notifiable condition diagnoses linked to NSW HIV diagnoses, 1993-2012, and rate of missed opportunities per 1000 population of people living with HIV (PLHIV), by Local Health District of residence at diagnosis**

| **Local Health District** | Notifiable Condition Diagnoses  (1993-2012)  (n) | Estimated Undiagnosed PLHIV  (1993-2012) (person-years) | Estimated total population (1993-2012) (person-years) | Missed Opportunities per 1000 person-years (Undiagnosed PLHIV) | Missed Opportunities per 100,000 person-years (total population) |
| --- | --- | --- | --- | --- | --- |
| Sydney | 57 | 49047 | 10,838,404 | 1.2 | 0.5 |
| South Western Sydney | 31 | 8546 | 16,278,374 | 3.6 | 0.2 |
| South Eastern Sydney* | 231 | 30449 | 15,657,379 | 7.6 | 1.5 |
| Illawarra Shoalhaven | 7 | 5664 | 7,048,676 | 1.2 | 0.1 |
| Western Sydney | 23 | 15232 | 15,931,394 | 1.5 | 0.1 |
| Nepean Blue Mountains | 8 | 10236 | 6,401,794 | 0.8 | 0.1 |
| Northern Sydney | 22 | 20704 | 15,799,082 | 1.1 | 0.1 |
| Central Coast | 6 | 4891 | 5,902,515 | 1.2 | 0.1 |
| Hunter New England | 26 | 10884 | 16,103,013 | 2.4 | 0.2 |
| Northern NSW | 6 | 5210 | 5,244,047 | 1.2 | 0.1 |
| Mid North Coast | 7 | 4821 | 3,779,383 | 1.5 | 0.2 |
| Southern NSW | 3 | 2181 | 3,599,075 | 1.4 | 0.1 |
| Murrumbidgee | 5 | 2203 | 4,296,454 | 2.3 | 0.1 |
| Western NSW/Far West | 5 | 3542 | 5,524,125 | 1.4 | 0.1 |
| **Total** | 440 | 173,612 | 132,403,715 | 2.5 | 0.3 |

*Including St Vincent’s Health Network and Justice & Forensic Mental Health
