# Supplementary material for: Identifying missed clinical opportunities for the earlier diagnosis of HIV in Australia, a retrospective cohort data linkage study
Source: PLoS One. 2018 Dec 6;13(12):e0208323. doi: 10.1371/journal.pone.0208323 (PMC6283600; doi:10.1371/journal.pone.0208323)
Supplement: S3 Table — (DOCX) [file pone.0208323.s003.docx]

S3 Table

Number of NSW hospital admissions linked to NSW HIV diagnoses, 2000-2012 and rate of missed opportunities for HIV diagnosis per 1000 person-years for population of people living with HIV (PLHIV), by Local Health District of admission

| **Local Health District** | Admissions (2000-2012) (n) | Estimated Undiagnosed PLHIV (2000-2012) (person-years) | Estimated total population (2000-2012) (person-years) | Missed Opportunities for HIV diagnosis per 1000 person-years (Undiagnosed PLHIV) | Missed Opportunities for HIV diagnosis per 100,000 person years (total population) |
| --- | --- | --- | --- | --- | --- |
| Sydney | 148 | 33771 | 7,284,301 | 4.4 | 2.0 |
| South Western Sydney | 94 | 6051 | 10,940,409 | 15.5 | 0.9 |
| South Eastern Sydney* | 496 | 20623 | 10,523,049 | 24.1 | 4.7 |
| Illawarra Shoalhaven | 48 | 4063 | 4,737,292 | 11.8 | 1.0 |
| Western Sydney | 114 | 10366 | 10,707,210 | 11.0 | 1.1 |
| Nepean Blue Mountains | 41 | 6884 | 4,302,533 | 6.0 | 1.0 |
| Northern Sydney | 138 | 15153 | 10,618,285 | 9.1 | 1.3 |
| Central Coast | 50 | 3444 | 3,966,977 | 14.5 | 1.3 |
| Hunter New England | 162 | 7621 | 10,822,552 | 21.3 | 1.5 |
| Northern NSW | 28 | 3629 | 3,524,432 | 7.7 | 0.8 |
| Mid North Coast | 36 | 3339 | 2,540,057 | 10.8 | 1.4 |
| Southern NSW | 12 | 1622 | 2,418,875 | 7.4 | 0.5 |
| Murrumbidgee | 14 | 1659 | 2,887,571 | 8.4 | 0.5 |
| Western NSW/Far West | 21 | 2449 | 3,712,668 | 8.6 | 0.6 |
| **Total** | 1,415 | 120,675 | 88,986,212 | 11.7 | 1.6 |

*Including St Vincent’s Health Network and Justice & Forensic Mental Health
