# Supplementary material for: Identifying missed clinical opportunities for the earlier diagnosis of HIV in Australia, a retrospective cohort data linkage study
Source: PLoS One. 2018 Dec 6;13(12):e0208323. doi: 10.1371/journal.pone.0208323 (PMC6283600; doi:10.1371/journal.pone.0208323)
Supplement: S4 Table — (DOCX) [file pone.0208323.s004.docx]

S4 Table

Number of NSW ED presentations linked to NSW HIV diagnoses, 2005-2012, and rate of missed opportunities per 1000 person-years for people living with HIV (PLHIV), by Local Health District of ED presentation

| **Local Health District** | ED Presentations  (2005-2012)  (n) | Estimated Undiagnosed PLHIV  (2005-2012) (person-years) | Estimated total population (2005-2012) (person-year) | Missed Opportunities per 1000 person-years (Undiagnosed PLHIV) | Missed Opportunities per 100,000 person-years (total population) |
| --- | --- | --- | --- | --- | --- |
| Sydney | 358 | 21769 | 4,569,021 | 16.4 | 7.8 |
| South Western Sydney | 123 | 3906 | 6,862,286 | 31.5 | 1.8 |
| South Eastern Sydney* | 732 | 13114 | 6,600,501 | 55.8 | 11.1 |
| Illawarra Shoalhaven | 83 | 2671 | 2,971,429 | 31.1 | 2.8 |
| Western Sydney | 130 | 6706 | 6,716,014 | 19.4 | 1.9 |
| Nepean Blue Mountains | 68 | 4469 | 2,698,730 | 15.2 | 2.5 |
| Northern Sydney | 98 | 10158 | 6,660,237 | 9.6 | 1.5 |
| Central Coast | 63 | 2299 | 2,488,255 | 27.4 | 2.5 |
| Hunter New England | 194 | 5031 | 6,788,361 | 38.6 | 2.9 |
| Northern NSW | 43 | 2362 | 2,210,673 | 18.2 | 1.9 |
| Mid North Coast | 36 | 2181 | 1,593,231 | 16.5 | 2.3 |
| Southern NSW | 10 | 1115 | 1,517,221 | 9.0 | 0.7 |
| Murrumbidgee | 11 | 1131 | 1,811,207 | 9.7 | 0.6 |
| Western NSW/Far West | 38 | 1590 | 2,328,742 | 23.9 | 1.6 |
| **Total** | 1,991 | 78,503 | 55,815,907 | 25.4 | 3.6 |

*Including St Vincent’s Health Network and Justice & Forensic Mental Health Network
